# Hypocholesterolemic Effects of Probiotic Mixture on Diet-Induced Hypercholesterolemic Rats

**DOI:** 10.3390/nu9030293

**Published:** 2017-03-16

**Authors:** Shang-Jin Kim, Sang Hoon Park, Hong-Sig Sin, Seung-Hwan Jang, Sang-Wang Lee, Seon-Young Kim, Bora Kwon, Kang-Yeol Yu, Su Young Kim, Dong Kwon Yang

**Affiliations:** 1 Department of Veterinary Pharmacology and Toxicology, College of Veterinary Medicine, Biosafety Research Institute and Korea Zoonosis Research Institue, Chonbuk National University, Iksan 54596, Korea; abbasj@jbnu.ac.kr; 2 Huvet Co. Ltd., Iksan 54541, Korea; hyunmu123@hanmail.net; 3 Chebigen Co. Ltd., Jeonju 54853, Korea; shsdo@hanmail.net (H.-S.S.); sampo999@hanmail.net (S.-H.J.); cbg31@chebigen.com (S.-W.L.); 4 Jeonju Agro-Biomaterials Institute, Jeonju 54810, Korea; seon02@jami.re.kr (S.-Y.K.); jasmin0207@jami.re.kr (B.K.); kangyu@jami.re.kr (K.-Y.Y.); zcbmmnn@jami.re.kr (S.Y.K.)

**Keywords:** hypercholesterolemia, high-cholesterol diet, probiotics mixture, *Bifidobacterium longum*, *Bifidobacterium lactis*, *Bifidobacterium breve*, *Lactobacillus reuteri*, *Lactobacillus plantarum*, liver steatosis

## Abstract

Growing evidence has indicated that supplementation with probiotics improves lipid metabolism. We aimed to investigate the beneficial effects of a probiotics mixture (PM) of three strains belonging to the species *Bifidobacterium* (*B. longum*, *B. lactis*, and *B. breve*) and two strains belonging to the species *Lactobacillus* (*L. reuteri* and *L. plantarum*) on cholesterol-lowering efficacy in hypercholesterolemic rats. A hypercholesterolemic rat model was established by feeding a high-cholesterol diet for eight weeks. To test the effects of PM on hypercholesterolemia, hypercholesterolemic rats were assigned to four groups, which were treated daily with low (1.65 × 10^9^ cfu/kg), medium (5.5 × 10^9^ cfu/kg), or high (1.65 × 10^10^ cfu/kg) doses of probiotic mixture or simvastatin for eight weeks. Significant reductions of serum total cholesterol (TC), triacylglycerol (TG), and low-density lipoprotein (LDL)-cholesterol levels, but increases of high-density lipoprotein (HDL)-cholesterol were observed after supplementation of PM in hypercholesterolemic rats. In PM-supplemented hypercholesterolemic rats, hepatic tissue contents of TC and TG also significantly decreased. Notably, the histological evaluation of liver tissues demonstrated that PM dramatically decreased lipid accumulation. For their underlying mechanisms, we demonstrated that PM reduced expressions of cholesterol synthesis-related proteins such as sterol regulatory element-binding protein 1 (SREBP1), fatty acid synthase (FAS), and acetyl-CoA carboxylase (ACC) in the liver. Taken together, these findings suggest that PM has beneficial effects against hypercholesterolemia. Accordingly, our PM might be utilized as a novel therapeutic agent for the management of hypercholesterolemia.

## 1. Introduction

Hypercholesterolemia is a risk factor of cardiovascular disease (CVD), type 2 diabetes mellitus, and metabolic syndrome [1]. The WHO has predicted that up to 40% of all deaths will be related to CVD by 2030, affecting approximately 23.6 million people around the world [2]. Indeed, the risk of heart attack is three times higher in patients with hypercholesterolemia than those who have normal blood lipid contents. Furthermore, a 1% increase in serum cholesterol concentration results in 2%–3% increase in the occurrence of CVD [3]. In addition, it is a major cause of atherosclerosis and atherosclerosis-associated diseases such as coronary disease and peripheral vascular disease [4]. It is characterized by high levels of total cholesterol (TC), low-density lipoprotein (LDL)-cholesterol, and triacylglycerol (TG), but low levels of high-density lipoprotein (HDL)-cholesterol in the blood vessels [5]. Hypercholesterolemia is also believed to be a crucial factor in the development of non-alcoholic fatty liver disease (NAFLD) [6]. The excess of TG by hypercholesterolemia can be stored as lipid droplets in the liver. Furthermore, excessive accumulation of TG within hepatocytes causes NAFLD [7]. NAFLD is considered as the most common causes of liver injury, and often occurs along with diabetes and obesity [8]. It can further progress to severe liver diseases such as liver fibrosis, cirrhosis, and end stage liver failure, and rarely hepatocellular carcinoma [9,10,11].

Probiotics are defined as “Live microorganisms which when administered in adequate amounts confer a health benefit on the host” by the Expert Panel commissioned in 2001 by the Food and Agriculture Organization of the United Nations, and supported by the World Health Organization [12]. Probiotics could improve gut health by inhibiting the growth and attachment of harmful bacteria [13]. In addition to improving gut health, probiotics have also been reported to exert to other health-promoting effects against many kinds of diseases, including hypertension [14], cancer [15], allergic symptoms [16], arthritis [17], hyperlipidemia [18], and so on. In particular, probiotics have also been studied for their cholesterol-lowering effects in animal and human studies. Among them, Lactobacilli and Bifidobacteria are well studied in terms of their cholesterol-lowering effects. *Lactobacillus acidophilus* reduces blood cholesterol by breakdown of cholesterol and de-conjugation of bile salts [19]. Another study has demonstrated that *Bifidobacterium longum* BL1 decreases serum TC, LDL-cholesterol, and TG, and increases HDL-cholesterol in humans [20]. However, certain strains of probiotics have demonstrated cholesterol-lowering properties while some strains have not. Furthermore, evidence and proposed mechanisms targeting cholesterol-lowering effects remain controversial.

In the present study, we investigated cholesterol-lowering effects of five potential probiotic strains (*Bifidobacterium longum* CBG-C11, *Bifidobacterium lactis* CBG-C10, *Bifidobacterium breve* CBG-C2, *Lactobacillus reuteri* CBG-C15, and *Lactobacillus plantarum* CBG-C21) on high cholesterol diet (HCD)-induced hypercholesterolemic rats. In addition, we also evaluated potential mechanisms underlying their cholesterol-lowering effect.

## 2. Materials and Methods

### 2.1. Bacteria and Culture

Each probiotic strain (*Bifidobacterium longum* CBG-C11, *Bifidobacterium lactis* CBG-C10, *Bifidobacterium breve* CBG-C2, *Lactobacillus reuteri* CBG-C15, and *Lactobacillus plantarum* CBG-C21) were obtained from Chebigen Co. Ltd. (Jeonju, Korea) and grown in Man Rogosa Sharpe (MRS) medium containing 0.5 mg/mL linoleic acid at 37 °C for 15 h. Cells were collected by centrifugation at 4000× *g* for 10 min at 4 °C. The bacteria were mixed with freezing conservative containing 10% creaming powder, 10% dextran, and 5% sorbitol and lyophilized. The lyophilized formulation containing 5.5 × 10^10^ cfu/g of five probiotic strains was used in animal study. The viabilities of the administered strains were confirmed.

### 2.2. Animals and Study Design

All animal experiments in this study were approved by the Animal Care Committee of Wonkwang University (Approval number: WKU16-24) and were performed according to the guidelines from the Wonkwang University IACUC the NIH principles for the Care and Use of Laboratory Animals. Male Sprague-Dawley rats aged 7 weeks (Samtako Biokorea, Daejeon, Korea) were used for all experiments. Rats were individually housed in cages maintained at 23 ± 2 °C with 50% ± 5% humidity and subjected to a 12 h light/dark cycle, and fed with either standard food diet (Std) or high cholesterol diet (HCD). HCD was prepared according to Paigen atherogenic diet (1.25% cholesterol, 0.5% cholate, and 15% fat) [21] for 8 weeks. After 1 week of acclimation, rats were divided into six groups: (1) control group treated with saline; (2) HCD-fed group; (3) HCD-fed + low dose 1.65 × 10^9^ cfu/kg/day probiotics mixture (PM); (4) HCD-fed + medium dose (5.5 × 10^9^ cfu/kg/day) PM; (5) HCD-fed + high dose (1.65 × 10^10^ cfu/kg/day) PM; (6) HCD-fed + 4 mg/kg/day simvastatin (as a positive drug which is commonly used for the treatment of hypercholesterolemia) treatment (*n* = 10 in each group). In the control group, rats were administered saline daily by oral gavage. For PM and simvastatin supplements, rats were administered indicative doses of PM or simvastatin daily by oral gavage. The animals were weighed and the remaining foods were weighed weekly to calculate food intake.

### 2.3. Measurement of Serum Lipids and Alanine Transaminase (ALT) and Aspartate Transaminase (AST)

At the end of 8 weeks, all rats were sacrificed after 12 h fasting and blood was then collected from the abdominal vein by heparinized syringe. Serum was collected by centrifugation at 1500× *g* for 15 min. A Hitachi 7020 system (Hitachi, Tokyo, Japan) was used for analysis of serum TC, TG, LDL-, and HDL-cholesterol levels.

### 2.4. Liver Histological Analysis

Liver tissues were carefully removed, rinsed, and fixed with 10% formalin solution and embedded in paraffin. Five micrometer sections were cut and stained with hematoxylin and eosin (H & E) and examined by light microscopy. The evaluation of liver steatosis score was conducted according to the previous study [22].

### 2.5. Measurement of Hepatic TC and TG

The hepatic lipids were extracted from the liver tissue using a chloroform/methanol mixed solution (2:1, *v*/*v*). Samples were then centrifuged at 12,000× *g* for 10 min. After obtaining supernatants, hepatic TC and TG levels were quantified by using commercial enzymatic kits (Asan Pharmaceutical Co., Asan, Korea).

### 2.6. Western Blot Analysis

Liver tissues were homogenized in RIPA lysis buffer containing protease inhibitor cocktail (Roche, Indianapolis, IN, USA). Protein homogenates were separated on SDS-PAGE gels and transferred to polyvinylidene difluoride (PVDF) membranes. After blocking for 1 h with 5% non-fat dry milk, the membranes were incubated overnight at 4 °C with antibodies against sterol regulatory element-binding protein 1 (SREBP1), fatty acid synthase (FAS), acetyl-CoA carboxylase (ACC), and *β*-actin (Santa Cruz Biotechnology, Dallas, TX, USA). Next, the membranes were incubated with the appropriate horseradish peroxidase (HRP)-conjugated secondary antibodies (Cell Signaling Tech, Beverly, MA, USA) for 1 h, and the bands were detected using enhanced chemiluminescence. The blots were scanned by a Bio-Rad ChemiDoc XRS and the intensity of each protein was quantified by Quantity One 4.5.0 software (Bio-Rad, Hercules, CA, USA).

### 2.7. Statistical Analysis

All data are reported as the mean ± SEM. Statistical significance was analyzed by using repeated measures or one-way ANOVA with Bonferroni post-hoc test (Prism 5.0.3, GraphPad Software Inc., San Diego, CA, USA). A *p*-value < 0.05 was considered statistically significant.

## 3. Results

### 3.1. Effects of Probiotics Mixture on Hypercholesterolemic Rats

**Figure 1:**
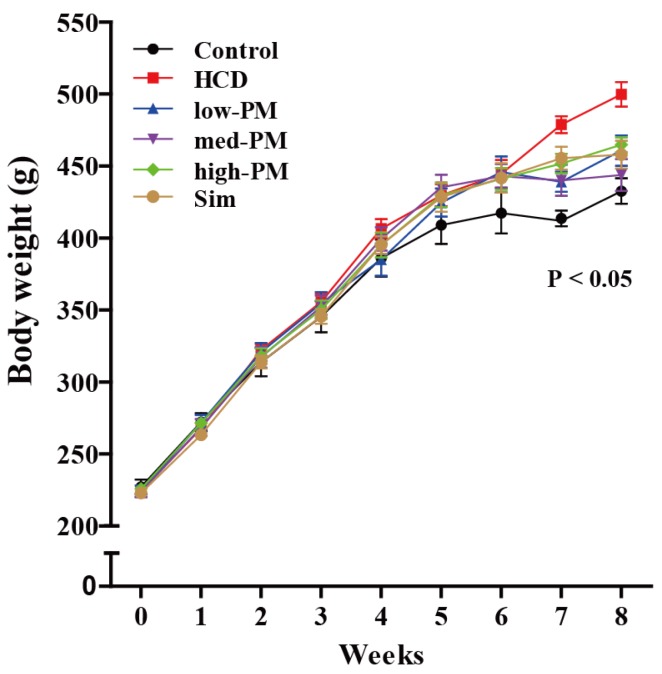
Body weight (BW) changes during experimental procedure. Rats were divided into control, high cholesterol diet (HCD)-fed, and PM-treated groups (*n* = 10 per group). BW was measured once a week in each group. *p*-value for ANOVA for repeated measures is given. HCD, high cholesterol diet-fed group; low-, med-, and high-PM: low (1.65 × 10^9^ cfu/kg/day), medium (5.5 × 10^9^ cfu/kg/day), and high doses (1.65 × 10^10^ cfu/kg/day) of probiotic mixture-treated group, respectively.

Figure 1 shows the growth curves on body weight changes during the experimental period. Body weight was steadily increased in each group. At 8 weeks, the body weight was significantly increased in HCD-fed group compared with control group (15.5% increase vs. control group). However, no difference in food intakes was observed between HCD-fed and control groups. No differences in food and water intakes were observed in probiotics mixture (PM)-supplemented groups (low, medium, and high doses, respectively) and simvastatin-treated group compared with control group (Table 1).

### 3.2. Effects of Probiotics Mixture on Serum Lipid Levels in Hypercholesterolemic Rats

**Figure 2:**
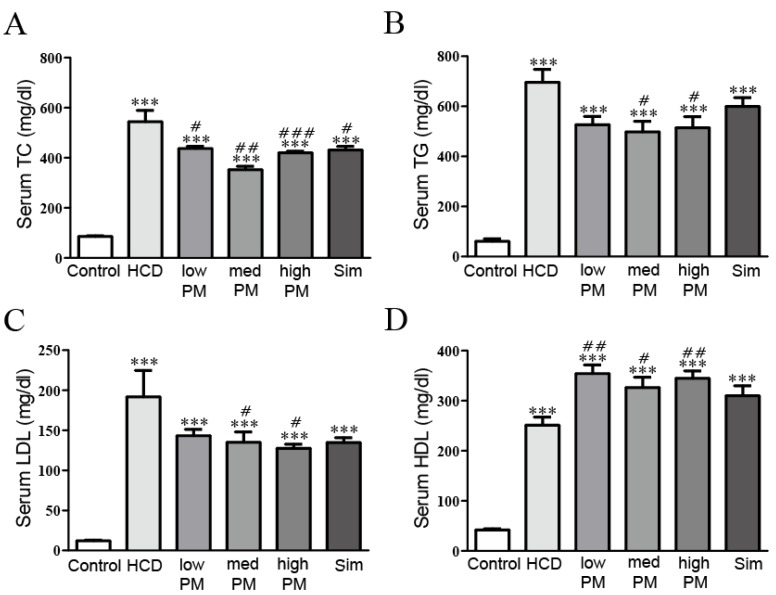
Effects of PM treatment on serum levels of (**A**) TC, (**B**) TG, (**C**) LDL-cholesterol, and (**D**) HDL-cholesterol in hypercholesterolemic rats. Data are expressed as mean ± SEM. Significance was measured by performing a one-way ANOVA followed by Bonferroni’s post-hoc test. *** *p* < 0.005 vs. control. ^#^ *p* < 0.05, ^##^ *p* < 0.01, ^###^ *p* < 0.005 vs. HCD-fed group. HCD, high cholesterol diet-fed group; low-, med-, and high-PM: low (1.65 × 10^9^ cfu/kg/day), medium (5.5E × 10^9^ cfu/kg/day), and high doses (1.65 × 10^10^ cfu/kg/day) of probiotic mixture-treated group, respectively; Sim, simvastatin-treated group; TC, total cholesterol; TG, triglycerides; LDL, low-density lipoprotein cholesterol; HDL, high-density lipoprotein cholesterol.

At 8 weeks after PM treatment, serum TC, TG, LDL-, and HDL-cholesterol in the HCD-fed group were significantly elevated compared with control group (6.4-fold, 11.4-fold, 16.0-fold, and 6.0-fold increases for TC, TG, LDL-, and HDL-cholesterol levels, respectively, vs. control group). Otherwise, PM treatment dramatically attenuated elevated levels of these lipid parameters compared with the HCD-treated group. TC level was significantly lower by 1.2-fold, 1.5-fold, and 1.3-fold in low, medium, and high doses of PM-treated groups, respectively, compared to the HCD-treated group. TG level was dramatically inhibited, by 1.32-fold, 1.4-fold, and 1.4 fold in low, medium, and high doses of PM-treated groups, respectively, compared to the HCD-treated group. LDL-cholesterol level was inhibited by 1.3-fold, 1.4-fold, and 1.5 fold in low, medium, and high doses of PM-treated groups, respectively, compared to the HCD-treated group. On the contrary, HDL-cholesterol level was increased by 1.4-fold, 0.9-fold, and 1.1 fold in in low, medium, and high doses of PM-treated groups, respectively compared to the HCD-treated group. Simvastatin (as a hypercholesterolemia drug) treatment has similar patterns with PM-treatment (Figure 2). Thus, PM supplementation could inhibit increased serum lipids under HCD-induced hypercholesterolemia.

### 3.3. Effects of Probiotics Mixture on Hepatic Steatosis in Hypercholesterolemic Rats

**Figure 3:**
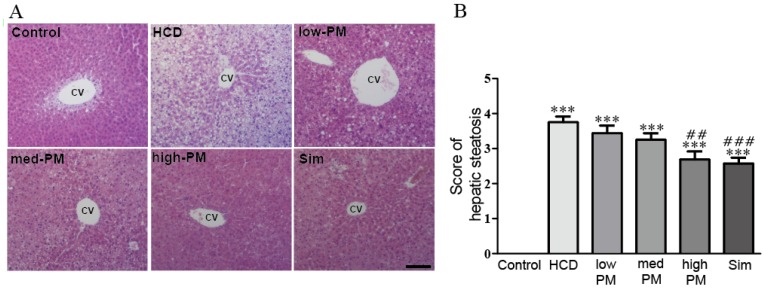
Effects of PM on hepatic steatosis in hypercholesterolemic rat. (**A**) Histological analysis of liver tissue in hypercholesterolemic rats. Liver sections were stained with hematoxylin and eosin (H & E) and examined under a light microscope (*n* = 8 per group); (**B**) Scores of hepatic steatosis of hypercholesterolemic rat livers. Scores were determined according to hepatocytes containing lipid droplets (*n* = 8 per group). Data are expressed as mean ± SEM. Significance was measured by performing a one-way ANOVA followed by Bonferroni’s post-hoc test. *** *p* < 0.005 vs. control. ^##^ *p* < 0.01, ^###^ *p* < 0.005 vs. HCD-fed group. HCD, high cholesterol diet-fed group; low-, med-, and high-PM: low (1.65 × 10^9^ cfu/kg/day), medium (5.5 × 10^9^ cfu/kg/day), and high doses (1.65 × 10^10^ cfu/kg/day) of probiotic mixture-treated group, respectively; Sim, simvastatin; CV, central vein. Scale bar, 50 μm.

Hypercholesterolemia is an important risk factor for NAFLD, which is characterized by steatosis, lobular inflammation, and hepatocellular ballooning [23]. To determine the effects of PM on NAFLD development in hypercholesterolemic rats, we examined the hepatic morphology in rats. As shown in Figure 3, while there was no obvious steatosis with clear hepatic cord and sinusoid in the control group, liver cells in the HCD-fed group exhibited massive fatty changes and severe steatosis with cytoplasmic vacuoles, many fat vacuoles, and microvesicles filled with small lipid droplets compared with control group. Otherwise, in PM-treated groups, the degrees of hepatic steatosis were remarkably improved compared with the HCD-fed group. Similarly, the simvastatin treatment also decreased the severity of hepatic steatosis by HCD (Figure 3). In addition, we also determined the score of hepatic steatosis according to the percentage of hepatocytes containing lipid droplets [19]. The score in the HCD-fed group was dramatically increased relative to the control group (3.75 score in HCD-fed group). Notably, treatment with probiotics mixture reduced the scores compared with HCD-fed group, with the most marked effect in the group treated with high dose of PM (3.35, 3.29, and 2.5 scores in low, medium, and high doses of PM-treated groups, respectively). A similar result was found for simvastatin treatment, with the score reduced to 2.5 (Figure 3).

### 3.4. Effects of Probiotics Mixture on Hepatic ALT, AST, TC, and TG in Hypercholesterolemic Rats

To determine whether PM treatment could mitigate the HCD-induced liver injury, the levels of hepatic TC and TG were examined. The levels of ALT and AST in the HCD-fed group were significantly higher than that of the control group (1.6-fold and 1.3-fold increases for ALT and AST, respectively, vs. control group). In contrast, hypercholesterolemic rats treated with PM or simvastatin had significantly attenuated levels of hepatic ALT and AST (64.4%, 59.2%, 79.2%, and 70.0% decreases for ALT; 71.3%, 60.2%, 78.9%, and 69.1% decreases for AST in low, medium, and high doses of PM, and simvastatin-treated groups, respectively, vs. HCD-fed group) (Table 2).

In addition, hepatic TG and TC levels in the HCD-fed group were also significantly higher than those of the control group (3.2-fold and 3.7-fold increases for TG and TC, respectively, vs. control group). In PM and simvastatin-treated groups, these levels were decreased compared to the HCD-fed group (91.6%, 83.3%, 63.8%, and 66.0% for TG; 94.0%, 89.6%, 88.5%, and 83.3% for TC in low, medium, and high doses of PM, and simvastatin-treated groups, respectively, vs. HCD-fed group) (Table 2). These data indicated that PM treatment improved the liver injury and hepatic lipid profiles in the hypercholesterolemic rats.

### 3.5. Effects of Probiotics Mixture on Expressions of Hepatic Cholesterol Homeostasis-Related Proteins in Hypercholesterolemic Rats

**Figure 4:**
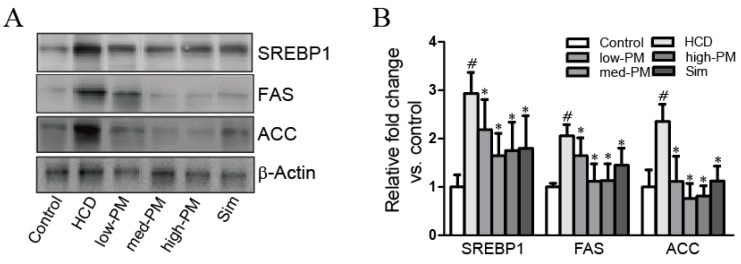
Effects of PM treatment on hepatic cholesterol homeostasis-related proteins in hypercholesterolemic rats. (**A**) Liver extracts from HCD diet, PM, and simvastatin treated groups were used for Western blot analysis; (**B**) Expression levels of SREBP1, FAS, and ACC were quantified by measuring band densities with National Institutes of Health (NIH) Image J software. β-actin was used as a loading control. Data are expressed as mean ± SEM. Significance was measured by performing a one-way ANOVA followed by Bonferroni’s post-hoc test. ^#^ *p* < 0.05 vs. control group. * *p* < 0.05 vs. HCD-fed group. HCD, high cholesterol diet-fed group; low-, med-, and high-PM: low (1.65 × 10^9^ cfu/kg/day), medium (5.5 × 10^9^ cfu/kg/day), and high doses (1.65 × 10^10^ cfu/kg/day) of probiotic mixture-treated group, respectively; Sim, simvastatin-treated group.

To explore the mechanisms underlying the effects of PM in hypercholesterolemia, expression levels of cholesterol homeostasis-related proteins such as SREBP1, FAS, and ACC were determined by Western blot analysis. The results showed that SREBP1 and its target proteins, FAS, and ACC in the HCD-fed group were significantly elevated compared with the control group (2.9-fold, 2.1-fold, and 2.4-fold increases for SREBP1, FAS, and ACC, respectively, vs. control group). In contrast, PM and simvastatin treatments significantly diminished the expression levels of these proteins in hypercholesterolemic rats (74.4%, 56.0%, 59.8%, and 61.3% decreases for SREBP1; 80.0%, 54.6%, 55.2%, and 70.6% decreases for FAS; 47.3%, 32.4%, 34.4%, and 47.7% decreases for ACC in low, medium, and high doses of PM, and simvastatin-treated groups, respectively, vs. HCD-fed group) (Figure 4).

## 4. Discussion

Hypercholesterolemia is a common cause for many diseases [24,25]. Therefore, intensive efforts have been made to develop lipid-lowering drugs, such as statins (e.g., pitavastatin, atorvastatin, simvastatin, etc.). Statins are most commonly used to reduce cholesterol levels, and are a well-established class of drugs in the treatment of hypercholesterolemia. Furthermore, statin drugs have been proven to improve liver functions by inhibiting enzymes involved in cholesterol synthesis in patients with hypercholesterolemia [26]. However, there are also reported side effects of statins, such as myopathy (the symptoms of which are muscle weakness and muscular pain) and rhabdomyolysis affecting 0.1%–0.2% of patients taking statins [27].

Therefore, the natural and safe properties of probiotics can be a useful strategy for the treatment of hypercholesterolemia. Among them, *Lactobacillus* and *Bifidobacterium* species have been well-studied on their hypolipidemic effects in animal and human studies. *L. plantarum* used as single or mixed with *L. paracasei* exhibited blood cholesterol lowering effect in high fat and cholesterol diet-fed rats [28]. *L. rhamnosus* hsryfm has beneficial effects on lipid metabolism in hyperlipidemic rats by regulating the gut microbiota [29]. Supplement of milk-yogurts fermented with *B. longum* could reduce about 50% TC, LDL-cholesterol, and TG concentrations in albino hypercholesterolemic rats. Another study also observed decreases of these lipid parameters after 4 weeks supplement of yogurt containing *B. longum* in humans [20].

Recently, some studies have attempted to elucidate their effects using multi-strain probiotics against diseases. A meta-analysis conducted by literature review showed that multiple-strain probiotics are more effective than a single strain in reducing NEC (necrotizing enterocolitis) and mortality in infants [30]. Probiotics mixture, which contains five strains, was effective in the treatment of NAFLD by ameliorating increased lipid profiles, liver function, and inflammatory markers [31].

We have screened the probiotic strains that have cholesterol-lowering effects by bile salt hydrolase (BSH) activity assay and cholesterol-removal assay, and found five bacteria having cholesterol-lowering effects, which were used for this study. In the present study, significant reductions in serum TC, TG, and LDL-cholesterol were observed after eight weeks of supplementation with probiotics mixture containing two lactobacilli (*L. reuteri* and *L. plantarum*) and three bifidobacteria (*B. longum*, *B. lactis*, and *B. breve*) strains in hypercholesterolemic rats (Figure 2). Indeed, since LDL-cholesterol is a major component of serum cholesterol, LDL-cholesterol level may be an important factor for reducing total cholesterol. Our data indicated that probiotic mixture is a good substance for treating hypercholesterolemia.

Generally, excess cholesterol in the body is deposited in hepatic cells and eventually causes NAFLD. As expected, we demonstrated that high-cholesterol diet induced increases of hepatic TC, TG, LDL-cholesterol, and liver injury markers such as ALT and AST in rats (Table 2). Moreover, hypercholesterolemia induced the liver steatosis—characterized by cytoplasmic vacuoles, many fat vacuoles, and microvesicles filled with small lipid droplets. Similar results were also shown in our liver histology (Figure 3). In contrast, hypercholesterolemic rats supplemented with the probiotics mixture displayed significant reductions in these hepatic lipid profiles and liver injury markers. In addition, our results from liver histology proved that supplementation with probiotics mixture had a potential effect in alleviating hepatic steatosis in hypercholesterolemic rats.

Several mechanisms by which probiotics affect hypercholesterolemia have been proposed: reduction of plasma cholesterol through reduction of the enterohepatic circulation of bile salts by bile salt hydrolase activity; reducing the bioavailability of cholesterol from the diet; and a decrease in systemic levels of blood lipids by inhibiting hepatic cholesterol synthesis [32]. In this study, we found that probiotics mixture ameliorated increased levels of SREBP1 and their target proteins, such as FAS and ACC in hypercholesterolemic rats (Figure 4). In previous report on hepatic steatosis, these proteins were found to be elevated in hepatic steatosis, implicating increased cholesterol and fatty acid biosynthesis as potential causes of lipid deposition. SREBP1 protein is an important liver transcription factor controlling many genes involved in the metabolism of cholesterol and other lipids [33]. Thus, our data indicated that probiotics mixture could lower the lipid levels via the inhibition of SREBP1-related lipid biosynthesis mechanism in the liver.

## 5. Conclusions

In conclusion, our results suggested that probiotic mixture of two lactobacilli and three bifidobacteria has the potential to reduce serum total cholesterol, triglycerides, and LDL-cholesterol levels in hypercholesterolemic rats. We also demonstrated that our probiotic mixture inhibited the hepatic steatosis and cholesterol synthesis signaling pathway in the liver. Therefore, our study provides that using this probiotic mixture is a potential strategy for the prevention of hypercholesterolemia.

## Figures and Tables

**Table 1:** Body weight (BW) and daily food and water intake during experimental procedure.

Parameter	Control	HCD	Low-PM	Med-PM	High-PM	Simvastatin
Initial BW (g)	227.2 ± 5.1	224.8 ± 2.6	224.3 ± 3.6	223.8 ± 3.6	226.0 ± 2.3	223.1 ± 2.2
Final BW (g)	432.7 ± 8.8	499.8 ± 8.5 ^#^	460.8 ± 10.5 *	443.8 ± 11.0 *	464.8 ± 5.1 *	458.0 ± 9.6 *
Food intake (g/day)	19.0 ± 0.5	18.37 ± 0.3	19.2 ± 0.6	19.1 ± 0.3	19.0 ± 0.5	18.9 ± 0.5
Water intake (g/day)	33.4 ± 1.5	28.0 ± 0.8	30.0 ± 1.7	31.2 ± 1.0	30.5 ± 0.7	27.5 ± 0.4

Data are expressed as mean ± SEM. Significance was measured by performing a one-way ANOVA followed by Bonferroni’s post-hoc test. ^#^ *p* < 0.05 vs. control. * *p* < 0.05 vs. HCD-fed group. HCD, high cholesterol diet-fed group; low-, med-, and high-PM: low (1.65 × 10^9^ cfu/kg/day), medium (5.5 × 10^9^ cfu/kg/day), and high doses (1.65 × 10^10^ cfu/kg/day) of probiotic mixture-treated group, respectively.

**Table 2:** Levels of hepatic ALT, AST, and lipids in hypercholesterolemic rats.

Parameter	Control	HCD	Low-PM	Med-PM	High-PM	Sim
ALT (IU/L)	72.3 ± 4.8	117.7 ± 18.5 ^##^	75.8 ± 17.9 *	69.7 ± 14.7 **	93.2 ± 10.7 *	82.3 ± 14.4 *
AST (IU/L)	136.0 ± 12.9	171.3 ± 18.1 ^#^	122.2 ± 13.9 **	103.2 ± 9.4 **	135.2 ± 11.9 *	118.4 ± 12.7 *
TG (mg/dL)	43.8 ± 4.3	140.3 ± 54.9 ^##^	128.6 ± 32.4 *	116.9 ± 34.5 *	89.54 ± 14.5 **	92.58 ± 16.6 *
TC (mg/dL)	41.6 ± 11.4	152.4 ± 27.1 ^##^	143.3 ± 32.2 *	136.5 ± 61.4 *	134.9 ± 46.8 *	127.0 ± 52.1 *

Data are expressed as mean ± SEM. Significance was measured by performing a one-way ANOVA followed by Bonferroni’s post-hoc test. ^#^ *p* < 0.05, ^##^ *p* < 0.01 vs. control. * *p* < 0.05, ** *p* < 0.01 vs. HCD-fed group. HCD, high cholesterol diet-fed group; low-, med-, and high-PM: low (1.65 × 10^9^ cfu/kg/day), medium (5.5 × 10^9^ cfu/kg/day), and high doses (1.65 × 10^10^ cfu/kg/day) of probiotic mixture-treated group, respectively; ALT, alanine transaminase; AST, aspartate transaminase; Sim, simvastatin-treated group; TG, triglycerides; TC, total cholesterol.

## References

[1] Gielen S., Landmesser U. (2014). The year in cardiology 2013: Cardiovascular disease prevention. Eur. Heart J..

[2] World Health Organization (WHO) (2009). Cardiovascular Disease; Fact. Sheet n°317.

[3] Davis C.E., Rifkind B.M., Brenner H., Gordon D.J. (1990). A single cholesterol measurement underestimates the risk of coronary heart disease. An empirical example from the Lipid Research Clinics Mortality Follow-up Study. JAMA.

[4] Tunstall-Pedoe H., Vanuzzo D., Hobbs M., Mahonen M., Cepaitis Z., Kuulasmaa K., Keil U. (2000). Estimation of contribution of changes in coronary care to improving survival, event rates, and coronary heart disease mortality across the WHO MONICA Project populations. Lancet.

[5] Dai F.J., Hsu W.H., Huang J.J., Wu S.C. (2013). Effect of pigeon pea (*Cajanus cajan* L.) on high-fat diet-induced hypercholesterolemia in hamsters. Food Chem. Toxicol..

[6] Browning J.D., Horton J.D. (2004). Molecular mediators of hepatic steatosis and liver injury. J. Clin. Investig..

[7] Matteoni C.A., Younossi Z.M., Gramlich T., Boparai N., Liu Y.C., McCullough A.J. (1999). Nonalcoholic fatty liver disease: A spectrum of clinical and pathological severity. Gastroenterology.

[8] Marchesini G., Brizi M., Bianchi G., Tomassetti S., Bugianesi E., Lenzi M., McCullough A.J., Natale S., Forlani G., Melchionda N. (2001). Nonalcoholic fatty liver disease: A feature of the metabolic syndrome. Diabetes.

[9] Younossi Z.M., Gramlich T., Liu Y.C., Matteoni C., Petrelli M., Goldblum J., Rybicki L., McCullough A.J. (1998). Nonalcoholic fatty liver disease: Assessment of variability in pathologic interpretations. Mod. Pathol..

[10] Bugianesi E., Leone N., Vanni E., Marchesini G., Brunello F., Carucci P., Musso A., De Paolis P., Capussotti L., Salizzoni M. (2002). Expanding the natural history of nonalcoholic steatohepatitis: From cryptogenic cirrhosis to hepatocellular carcinoma. Gastroenterology.

[11] Neuschwander-Tetri B.A., Caldwell S.H. (2003). Nonalcoholic steatohepatitis: Summary of an AASLD single topic conference. Hepatology.

[12] Food and Agriculture Organization (FAO)/World Health Organization (WHO) (2001). Evaluation of Health and Nutritional Properties of Powder Milk and Live Lactic Acid Bacteria.

[13] Holzapfel W.H., Schillinger U. (2002). Introduction to pre- and probiotics. Food Res. Int..

[14] Yeo S.K., Liong M.T. (2010). Angiotensin I-converting enzyme inhibitory activity and bioconversion of isoflavones by probiotics in soymilk supplemented with prebiotics. Int. J. Food Sci. Nutr..

[15] Hirayama K., Rafter J. (2000). The role of probiotic bacteria in cancer prevention. Microbes Infect..

[16] Weston S., Halbert A., Richmond P., Prescott S.L. (2005). Effects of probiotics on atopic dermatitis: A randomised controlled trial. Arch. Dis. Child..

[17] Baharav E., Mor F., Halpern M., Weinberger A. (2004). Lactobacillus GG bacteria ameliorate arthritis in lewis rats. J. Nutr..

[18] Kumar M., Rakesh S., Nagpal R., Hemalatha R., Ramakrishna A., Sudarshan V., Ramagoni R., Shujauddin M., Verma V., Kumar A. (2013). Probiotic *Lactobacillus rhamnosus* GG and Aloe vera gel improve lipid profiles in hypercholesterolemic rats. Nutrition.

[19] Anderson J.W., Gilliland S.E. (1999). Effect of fermented milk (yogurt) containing *Lactobacillus acidophilus* L1 on serum cholesterol in hypercholesterolemic humans. J. Am. Coll. Nutr..

[20] Xiao J.Z., Kondo S., Takahashi N., Miyaji K., Oshida K., Hiramatsu A., Iwatsuki K., Kokubo S., Hosono A. (2003). Effects of milk products fermented by *Bifidobacterium longum* on blood lipids in rats and healthy adult male volunteers. J. Dairy Sci..

[21] Paigen B., Morrow A., Brandon C., Mitchell D., Holmes P. (1985). Variation in susceptibility to atherosclerosis among inbred strains of mice. Atherosclerosis.

[22] Kleiner D.E., Brunt E.M., Van Natta M., Behling C., Contos M.J., Cummings O.W., Ferrell L.D., Liu Y.C., Torbenson M.S., Unalp-Arida A. (2005). Design and validation of a histological scoring system for nonalcoholic fatty liver disease. Hepatology.

[23] Brunt E.M., Kleiner D.E., Wilson L.A., Belt P., Neuschwander-Tetri B.A., Network N.C.R. (2011). Nonalcoholic fatty liver disease (NAFLD) activity score and the histopathologic diagnosis in NAFLD: Distinct clinicopathologic meanings. Hepatology.

[24] Artham S.M., Lavie C.J., Milani R.V., Ventura H.O. (2008). The obesity paradox: Impact of obesity on the prevalence and prognosis of cardiovascular diseases. Postgrad. Med..

[25] Bellentani S., Saccoccio G., Masutti F., Croce L.S., Brandi G., Sasso F., Cristanini G., Tiribelli C. (2000). Prevalence of and risk factors for hepatic steatosis in northern Italy. Ann. Intern. Med..

[26] Vaughan C.J., Murphy M.B., Buckley B.M. (1996). Statins do more than just lower cholesterol. Lancet.

[27] Bellosta S., Paoletti R., Corsini A. (2004). Safety of statins: Focus on clinical pharmacokinetics and drug interactions. Circulation.

[28] El-Shafie H.A., Yahia N.I., Ali H.A., Khalil F.A., El-Kady E.M., Moustafa Y.A. (2009). Hypocholesterolemic action of *Lactobacillus plantarum* NRRL-B-4524 and lactobacillus paracasei in mice with hypercholesterolemia induced by diet. Aust. J. Basic Appl. Sci..

[29] Chen D., Yang Z., Chen X., Huang Y., Yin B., Guo F., Zhao H., Zhao T., Qu H., Huang J. (2014). The effect of *Lactobacillus rhamnosus* hsryfm 1301 on the intestinal microbiota of a hyperlipidemic rat model. BMC Complement. Altern. Med..

[30] Chang H.Y., Chen J.H., Chang J.H., Lin H.C., Lin C.Y., Peng C.C. (2017). Multiple strains probiotics appear to be the most effective probiotics in the prevention of necrotizing enterocolitis and mortality: An updated meta-analysis. PLoS ONE.

[31] Al-Muzafar H.M., Amin K.A. (2017). Probiotic mixture improves fatty liver disease by virtue of its action on lipid profiles, leptin, and inflammatory biomarkers. BMC Complement. Altern. Med..

[32] Pereira D.I., Gibson G.R. (2002). Effects of consumption of probiotics and prebiotics on serum lipid levels in humans. Crit. Rev. Biochem. Mol. Biol..

[33] Shimomura I., Bashmakov Y., Horton J.D. (1999). Increased levels of nuclear SREBP-1c associated with fatty livers in two mouse models of diabetes mellitus. J. Biol. Chem..

